# How to design a ROCI (Response Over Continuous Intervention) randomised trial: guidance and a case study

**DOI:** 10.1186/s12874-026-02786-4

**Published:** 2026-04-25

**Authors:** Matteo Quartagno, Ehsan Ghorani, Tim P. Morris, Henry Bern, Michelle N. Clements, A. Sarah Walker, James R. Carpenter, Ian W. White, Koen B. Pouwels, Michael J. Seckl, Mahesh KB Parmar

**Affiliations:** 1 MRC Clinical Trials Unit, Institute of Clinical Trials and Methodology, University College London, 90 High Holborn, Second Floor, London, WC1V 6LJ UK; 2 Department of Medical Statistics, Faculty of Epidemiology and Population Health, London School of Hygiene and Tropical Medicine, London, UK; 3 Department of Medical Oncology, Charing Cross Hospital Campus of Imperial College London, London, UK; 4 Nuffield Department of Clinical Medicine, University of Oxford, Oxford, UK; 5 Nuffield Department of Primary Care Health Sciences, University of Oxford, Oxford, UK

**Keywords:** Clinical trials, ROCI, Duration design, Sample size, Non-inferiority

## Abstract

**Background:**

An important challenge in late-phase drug development is selection of the optimal parameters for treatment administration, including dose, duration and frequency. Whilst these are (near-)continuous parameters, conventional non-inferiority trial designs are not well suited to explore multiple options across a range of values. The ROCI (Response Over Continuous Intervention) randomised trial design is an alternative to standard non-inferiority trials, addressing limitations in selecting optimal treatment regimens, but practical guidance in its application is limited.

**Methods:**

We outline key design considerations for ROCI trials, including modelling the treatment–response relationship, selecting treatment levels and arms, defining optimality criteria, and determining power and sample size. A flexible fractional polynomial approach is recommended to estimate the treatment–response relationship, and two power definitions—optimal and acceptable—are considered. We apply these principles to REFINE-Lung, which investigates whether extended pembrolizumab dosing intervals in advanced non-small cell lung cancer (NSCLC) maintain efficacy while reducing unnecessary overtreatment. Sample size calculations use simulation-based methods or an analytic formula for binary outcomes.

**Results:**

REFINE-Lung was designed with five administration frequency arms, exploring the range within which the optimal frequency is likely to exist (6-, 9-, 12-, 15-, and 18-weekly dosing), with the primary endpoint 2-year overall survival. Initial sample size calculations targeted 1,750 patients to provide 80% optimal power, but in order to accelerate completion this was adjusted to 1,100 patients targeting acceptable power. An interim analysis strategy allowed adaptation based on early data on progression-free survival. This approach balances statistical robustness with feasibility while ensuring ethical equipoise in dose de-escalation.

**Conclusions:**

The ROCI design is an efficient alternative to standard non-inferiority trials for optimising continuous aspects of treatment administration. By sharing information across multiple treatment levels and leveraging flexible modelling approaches, ROCI trials improve efficiency and reliability in identifying optimal treatment regimens. The REFINE-Lung trial case study demonstrates applying these principles, providing a framework for future trials aiming to refine treatment administration strategies.

## Introduction

Most late-phase clinical trials are designed to compare two different treatments, one typically representing the standard-of-care; the variable representing the two treatments is then binary. In some situations, instead, we want to find the optimal value for some aspect of treatment administration, like duration, dose or frequency of administration which varies across a continuum: a standard trial might choose to compare arbitrary single or multiple values vs. control. This is common in early drug development phases where new compounds undergo phase I and/or II studies to select a dose for later phases, with a correspondingly vast literature on dose-finding methods [1].

Sometimes, however, optimisation of treatment administration is required at later phases, for example where treatments are already known to be effective clinically, but early-phase trials did not optimise some aspects of the treatment regimen, meaning evidence for currently recommended guidelines is scarce or inconsistent. The definition of ‘optimisation’ varies between applications: in some scenarios, we might simply want to maximise efficacy, but often we want the least intensive administration routine (the minimum duration/dose/frequency) leading to acceptable treatment efficacy. Here, the goal is to reduce overall treatment administration compared to the current standard-of-care. Reasons include reducing severe side-effects (e.g. cancer immunotherapy), limiting the spread of antimicrobial resistance (e.g. antibiotics) or improving adherence (e.g. novel TB treatments). Since it is essential to preserve efficacy while reducing overall treatment intensity, the standard randomised trial design addressing this problem has been a two-arm non-inferiority design comparing the current standard-of-care with a single reduced schedule.

A critical issue with this two-arm non-inferiority design is that if the experimental arm (e.g., shorter duration or lower dose than standard-of-care) is chosen poorly, the trial will inevitably conclude inferiority even though an optimal non-inferior administration routine exists. The ROCI (Response Over Continuous Intervention) randomised trial design was proposed [2] as a practical alternative, originally in the specific setting of reducing antibiotic treatment duration. It tackled this issue by (i) randomising patients to multiple points along the continuum, increasing the chance of including the “optimal” research arm(s), or at least covering it within the experimental arms, and (ii) modelling the continuous duration–response relationship, sharing information across durations instead of treating them as independent, thus improving efficiency. Given the motivation to reduce duration, ROCI was initially DURATIONS [4]; however, it can be applied to any continuous treatment administration variable where the goal is to find its optimal value. For example, it can also be used to optimise treatment administration frequency, as in our REFINE-Lung case study discussed in detail below. To reflect its flexibility to optimise variables beyond treatment duration, we hereafter refer to the design as ROCI (Response Over Continuous Intervention); if the design incorporates adaptation, it can be considered a Multi-Arm Multi-Stage (MAMS) design [3], and hence referred to as MAMS-ROCI.

Parallel-arm trials address a simple binary question (e.g. is A non-inferior to B?) and are designed to control type I error and minimise type II error. The main question answered by ROCI trials is instead continuous in nature, as we already know that at least the standard treatment administration is effective compared to no treatment, and we are interested in finding the optimum (e.g. the lowest acceptable, or the most effective) in a pre-specified range. The relevant measures of performance for a ROCI trial (i.e. the equivalents of power and type I error rate) are therefore less clear, complicating the design.

In Quartagno et al. (2018) [2], a hypothetical trial was designed focusing on a novel performance measure, the probability of a trial estimating the duration–response relationship within a pre-specified absolute error. Simulations showed that a sample size of 500 patients divided between five to seven duration arms captured the duration–response relationship well in the scenarios considered, motivated by an antibiotic trial, irrespective of the shape of the relationship. Such a performance measure is suitable when the outcome of the trial is the whole curve. However, in practice, researchers would use the estimated relationship to make informed decisions. Therefore, subsequently Quartagno et al. (2020) [4] compared the properties of different methods of drawing inference from the duration–response relationship, finding that an inferential strategy based on estimating the shortest duration non-inferior to control within a certain margin works well, particularly if using non-parametric bootstrap to handle uncertainty.

Supposing that a researcher wishes to plan a ROCI randomised trial, using the estimated response relationship and non-parametric bootstrap analysis, how should they determine the most appropriate sample size, number and spacing of arms and other key design features? This paper answers this question, first giving guidelines on choices that statisticians should make and then illustrating their application using the REFINE-Lung trial.

The paper is organised as follows: in "Motivating trial example: REFINE-Lung" section, we introduce the problem faced in our case study, the REFINE-Lung trial; in "Design considerations" section, we set out design considerations for planning a (MAMS-)ROCI trial, in particular determining the sample size required to adequately power the trial; and "Implementation: REFINE-Lung" section illustrates these considerations in the REFINE-Lung design. We conclude with discussion and plans for future work in "Discussion" section.

## Motivating trial example: REFINE-Lung

Treatments that block immune inhibitory receptors of their ligands (checkpoint immunotherapies) reinvigorate the immune response to control cancer, and have transformed outcomes for patients with many cancers. For example, amongst patients with advanced non-small cell lung cancer (NSCLC), 5 year survival is approximately 30–40% with the immunotherapy agent pembrolizumab compared to 5–10% with chemotherapy, the previous standard-of-care.

The recommended pembrolizumab dose is 400 mg every 6 weeks, but evidence suggests this may be unnecessarily high, resulting in overtreatment [5]. Optimising administration frequency within a fixed time horizon is attractive given high drug costs, quality of life implications from more frequent hospital visits for drug administration and the potential to reduce side effects. The latter cause significant issues in up to 5% of patients receiving pembrolizumab, may be life-threatening and almost invariably require large doses of steroid sometimes over several months with their own associated health issues (e.g. PMID: 31944278).

It is not clear whether, and by how much, administration frequency can be reduced without significantly impacting efficacy. In a (MAMS-)ROCI trial, patients would be randomised to several treatment frequencies across a range, and the frequency-response relationship estimated to establish the optimal frequency that does not compromise efficacy vs. standard-of-care 6-weekly therapy.

Several practical questions arise. Which frequencies should the patients be randomised between? What should the total sample size be? Should we adopt an outcome-adaptive design?

## Design considerations

Suppose we aim to design a ROCI trial to optimise a certain aspect of treatment administration that is a continuous variable, $\:A$. For example, we may target dose, frequency and/or duration of treatment. The currently recommended treatment level is $\:{A}_{std}$, and we wish to investigate whether this could be reduced (or extended), down to $\:{A}_{min}$. How should a researcher approach the sample size calculation and, more generally, the design, of a specific randomised trial?

We list here a series of design considerations and give recommendations on how to tackle these. 

### Treatment-response relationship model

The first choice is the model for the treatment-response relationship; this is important as it affects other design decisions. The class of model will be determined by the outcome type (e.g. logistic regression for a binary outcome); we discuss how treatment intensity is entered into the model.

Where there is a clear clinical or pharmacological rationale, a specific model can be used; for example Emax [6] models for dose-response relationships. However, often no model is expected to work well a priori, for at least two reasons: (i) there might be no biological reason to assume a specific model and there may be little to no data available or (ii) a specific model might be potentially a good fit for an individual, but not at the population level.

In such cases, flexible regression methods are a good method of sharing information across arms without forcing non-evidence based relationships. We previously considered fractional polynomial and spline-based methods and, when used optimally, either strategy can be recommended. Fractional polynomial (FP) models [7] use several pre-defined polynomial transformations and choose the best one based on specific criteria. They are particularly appealing because implementation is easier and requires fewer decisions. However, there are several options, with different assumptions. For example:

1. Number of power terms: the simplest option is a single power term (FP1). Because A is always positive, with only one power, FP1 implies strict monotonicity, i.e. more treatment will consistently mean better (or worse) outcomes. It can therefore be used as a strategy only when this assumption is considered reasonable (given the primary endpoint) and, even then, it is slightly less flexible than other options using more powers. Using (up to) two powers is more flexible and does not force monotonicity. Fractional polynomials are rarely used with more than two powers, as any additional advantages in flexibility are outweighed by additional complexity.
2. Type of algorithm: there are at least two different ways FP can be implemented. The powers (one in FP1/two in FP2) can be forced into the model (termed “FP1-fixed”/“FP2-fixed”), by selecting the one/two powers that lead to the best fit (likelihood). Alternatively (termed “FP1-select”/“FP2-select”), powers are included only if there is evidence that they improve the model fit substantially versus the best fitting FP1 model (in the case of FP2) or to a simple linear model (in the case of FP1). Neither strategy affects the monotonicity assumption for FP1. While an FP2 model does not force monotonicity, if used with the “FP2-select” algorithm it strongly favours monotonicity, as the ”select” algorithm only selects two powers (allowing for non-monotonicity) if there is clear evidence that the second power improves the fit of the monotonic FP1 or linear models.

The “select” algorithm is the default in the mfp [8] package in R

**Fig. 1:**
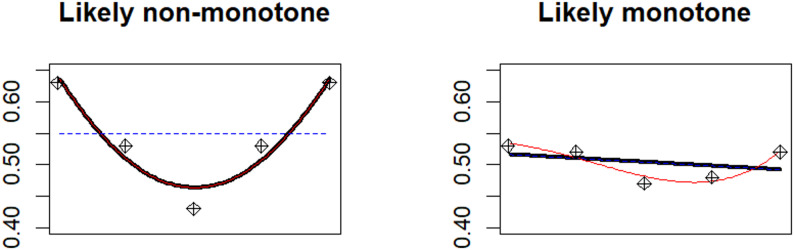
Left Panel: FP2-select (bold black) and FP2-fixed (red, superimposed) reflects a likely non-monotonic shape of the curve, while FP1-select is necessarily monotonic and does not fit well. Right panel: this time FP2-select matches FP1-select, as the non-monotonicity in the data (crosses) is likely just due to chance, while FP2-fixed is likely subject to overfitting

Therefore, the choice between the different methods should be driven by the plausibility of their assumptions. If the relationship can be assumed to be monotonic, “FP1-fixed” or “FP1-select” might work well. If monotonicity is regarded as plausible, but not certain, “FP2-select” might be the best option. If any shape is plausible, and there really is no expectation of monotonicity, “FP2-fixed” might be preferable instead. Figure 1 summarises this by giving an idea of why “FP2-select” might be preferable: it correctly selects a non-monotonic curve when there is clear evidence for it (left panel) matching “FP2-fixed”, while it sticks with the best FP1 when non-monotonicity is likely to be just due to random error (right panel).

Of note, we previously showed [4] that “FP2-fixed” is better than “FP2-select” at controlling type I error across different scenarios, where a trial commits type I error if it recommends a treatment level for which the treatment efficacy is beyond the associated non-inferiority margin (for example if it recommends a 8-day duration, for which the risk ratio against control was 1.5 and its non-inferiority margin was 1.25); however, this comes at the cost of increased sample size, and when the monotonicity assumption is likely to hold, the type I error implications are minimal. 

### Target scenarios for power

Standard sample size calculations for binary outcomes assume an expected event risk $\:{{\uppi\:}}_{0}$ in the control arm and estimate the sample size needed to detect a statistically significant difference with a given probability assuming that the experimental arm has event risk $\:{{\uppi\:}}_{1}$. For conventional two arm non-inferiority trials, often π1 = π0, as the alternative hypothesis is that both arms are equivalent. One option for ROCI designs is therefore to follow a similar strategy and target the power to detect non-inferiority under a flat treatment-response relationship, i.e. under the assumption that the treatment does not affect risk within the range considered. This is appealing from a patient perspective, as it implies that the goal is to identify treatments that are at least as effective as $\:{A}_{max}\:$by ensuring the trial is powered to reduce exposure only when this is not at the cost of effectiveness.

An alternative is to assume a non-flat treatment-response relationship, based on expert opinion and any other data sources. Since the aim of the ROCI design is to be as robust as possible to different potential relationships between treatment and response, this approach can be extended by calculating sample sizes under a range of plausible treatment-response scenarios, reflecting uncertainty about the true relationship. Having obtained all the different sample size estimates across scenarios, one could either take the maximum, to ensure a certain power is achieved irrespective of the true underlying scenario, or take the sample size whose associated average power across scenarios meets the predefined level.

### Number and position of arms

First, a researcher should define the range of treatment levels across which the clinical community would have equipoise. This will include the standard-of-care $\:{A}_{std}$ and the interval [$\:{A}_{min}$, $\:{A}_{max}$], with $\:{A}_{min}$ and $\:{A}_{max}$ defining the minimum and maximum treatment levels ($\:{A}_{max}$ or $\:{A}_{min}\:$, one of which will often be $\:{A}_{std}$). Adaptive steps can be used to avoid randomising patients directly into arms that are very different from the standard-of-care without first collecting data on the safety and efficacy of smaller reductions in levels (see "Adaptive steps: walking before running" section).

The choice of the specific arms then depends on the model for the treatment–response relationship. Where a specific model is justifiable, there is usually an optimal design. For example, where a linear model was considered highly likely, the best approach would be to simply randomise patients to two arms, $\:{A}_{min}$ and $\:{A}_{max}$, while with a quadratic model one should ideally add a third, intermediate arm.

However, as above, the ROCI design is most often used in the absence of strong evidence for a specific model, with flexible methods, such as fractional polynomials, used as the analysis model. For model fit to be satisfactory, it is necessary to have at least as many arms as parameters in the model (ideally at least one more). Therefore, with FP2, at least 5 arms are needed, as there are potentially five parameters: an intercept, up to two powers and the two corresponding coefficients. While 6–7 arms improves estimation of the relationship, and leads to smaller sample sizes per arm, the total sample size might increase with more arms [9]. Hence, at this stage, one could bring forward several suggestions for possible number of arms, and then the final decision, e.g. on whether to adopt five to seven arms, can be based on trade-offs, considering factors such as total sample size and its feasibility, and the clinical importance of including specific values along the continuum as arms to which patients are randomised.

Pragmatically, it is important to make sure that the selected arms reflect values that the community judges to be clinically usable. Furthermore, it is important that there is equipoise between all arms, i.e. all arms should be expected to be at least non-inferior to standard-of-care in the considered scenarios, such that patients are not exposed to potential preventable harm. For example, if the non-inferiority margin was set as a fixed 5% points difference across the administration continuum, none of the expected risks in the less intensive arms should be 5% points or more worse than in the control arm.

Finally, in the absence of any additional information about model form and feasibility, spacing arms equidistantly across [$\:{A}_{min}$, $\:{A}_{max}$] is a safe choice.

### “Optimality” criterion

Having chosen the arms, another important aspect to clarify in view of the sample size calculation is what we mean by “optimal” treatment value. This does not necessarily need to be one of the levels that patients are randomised to, because estimating the treatment-response relationship means we can infer any optimal treatment level within [$\:{A}_{min}$, $\:{A}_{max}$]. In our applications, the “optimal” value is typically defined as the minimal treatment level that is non-inferior to the standard-of-care or to the most intensive administration. This second option allows one to naturally avoid bio-creep [10], i.e. erosion of treatment efficacy by changing the standard-of-care multiple times, but might sometimes require larger sample sizes.

This criterion requires careful definition of “non-inferior”. First a precise estimand [11], must be chosen, discussing population-level summary measures, e.g. the risk difference or risk ratio, and strategies to handle intercurrent events. We can choose any summary measure irrespective of the model class chosen in 3.1, e.g. using logistic regression does not require an odds ratio as the summary measure [12]. Second, the non-inferiority margin should be defined. This may be different for different treatment levels, recognising different trade-offs for larger or smaller reductions. We term the curve that defines the non-inferiority margins over different treatment levels the “acceptability curve”.

While this optimality definition is the most used in our trials, it is by no means the only possible definition. The T4P trial (ISRCTN79371664, registration date: 30th September 2022) aims to detect the optimal platelet threshold below which to give platelet transfusions to patients admitted to intensive care units; the goal is to find the actual optimum in the relationship, so to simply maximise the primary outcome, i.e. superiority in survival risk. Another optimality option would be the point where the distance between observed outcome and non-inferiority margin is furthest.

### Power definitions

**Fig. 2:**
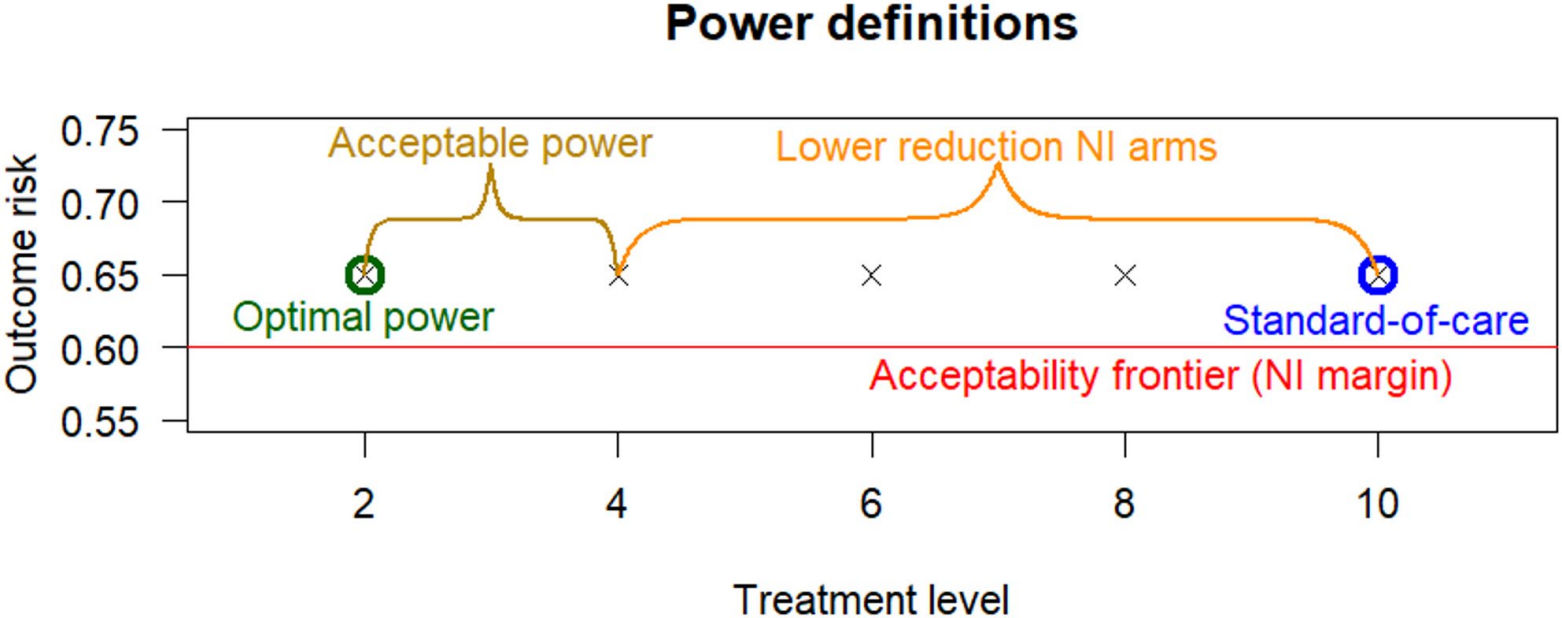
Alternative power definitions in a hypothetical scenario with arms at 2, 4, 6, 8 and 10. The crosses represent the hypothetical outcome risks at these arms (fixed at 65%). The red line represents the NI margin. All arms are NI. With optimal power we target the probability to recommend 2 days. With acceptable power we target the probability to recommend in the range [2,4]. The arms in (4, 10) are NI to 10, but are not considered for powering as they imply lower reduction

We previously proposed two possible power definitions for ROCI trials with discrete treatment levels: (i) *optimal power* and (ii) *acceptable power*. Optimal power is the probability that, in a specific, non-null, scenario, the trial recommends exactly *the* optimal treatment level, for example the shortest duration that is non-inferior to control. Acceptable power is the probability that, in the same scenario, a trial ends up recommending *an* acceptable (e.g. non-inferior to control) treatment level different from the control. These are depicted in Fig. 2.

Where treatment level is genuinely continuous rather than integerised, the concept of optimal power is challenging, since even if the optimum is 3.78 days say, the practical implications of identifying 3.67 days are minimal. On the other hand, optimal power is appealing if the treatment levels investigated are quite spread out and hence have different clinical significance. In such cases, one might feel that targeting the usual 80% or 90% levels for optimal power would be necessary to convince funders; however, this may be too ambitious in most circumstances because it requires very large sample sizes, and often multiple intensities will be clinically similar, hence even a trial recommending a treatment level slightly different from the optimal could be considered similarly successful. For these reasons, a lower level could be targeted for optimal power, e.g. 70% or anything that could be reasonably justified.

The sample sizes targeting equivalent levels of acceptable power will inevitably give less information. Whether or not a certain level of information is enough may depend on the specific way we define what is “acceptable”. The original definition of “acceptable” as “any arm which is non-inferior to control” may be too extreme, particularly in situations where treatment in one of the arms is very similar to treatment in the control arm (although this can be controlled by the choice of arms). Depending on the specific scenario, one could consider “acceptable” power as the probability of reducing treatment by at least 50% or by a certain fixed amount. In practice, in many cases, a good compromise could be to define “acceptable” as any treatment administration within a certain range from the optimal, e.g. an acceptable duration could be one which is within three days from the optimal one, under the provision that it is still non-inferior to control. Targeting standard levels of 80% (or 90% if feasible) would likely be more feasible with acceptable power, and this could help convince funders, although we argue there is no magic in the standard values generally used, and hence any other level could be targeted, if justified satisfactorily.

### Multiplicity adjustment

In trials that randomise over multiple arms it is typical to worry about multiple comparisons. In general, the goal of a ROCI trial is to estimate a single curve to find the least intensive treatment administration that is non-inferior to the control. This is equivalent to finding the point where the fitted treatment–response curve crosses the non-inferiority margin. Because it is a single question that needs to be answered, there is no need for adjustment for multiple comparisons. This is particularly clear in situations where, as is usually the case, one would not declare one treatment level as non-inferior if a more intense one was not found to be non-inferior; this has been labelled in the past a ‘gatekeeping strategy’ [13] and has been shown to control error rates acceptably [4].

This is particularly relevant in situations where a monotonic treatment-response relationship is considered likely at trial design, and aligns well with the existing literature on multiple comparisons and multiplicity adjustment [14–16].

### Sample size calculation method

Having decided on the parameters above, the statistician needs to perform the actual sample size calculation. Given the novelty and flexibility of the design, this was initially done using simulation, which is still a reasonable approach. However, planning and running simulations can take time, particularly when they include fitting several models and using bootstrap within each repetition, potentially for several different sample sizes. We recently proposed a sample size calculation approach that can work well under simple assumptions. This is based on generation of an expected outcome data set and estimation of the variances around the estimated population-level summary measures of interest (through use of bootstrap or simulations).

The formula for binary outcome is available as part of the R package {dani}, and we show how it can be used in "Implementation: REFINE-Lung" section.

### Adaptive steps: walking before running

In some situations, it is reasonable to be cautious about allowing patients to be randomised to treatment administrations that are far from the current standard-of-care at the beginning of trial recruitment. Equipoise about randomising to values far from standard-of-care may only be achieved when initial data from values closer to standard-of-care are reassuring. In such situations, researchers could adopt Multi-Arm, Multi-Stage (MAMS) methods and extend them to ROCI designs, using adaptive steps to modify the design while information is collected. For example, they might initially randomise only to two arms, the standard-of-care and a moderately reduced arm, and move to a larger ROCI design in absence of any lack-of-efficacy signal.

While it would theoretically be possible to use more general outcome-adaptive randomisation methods, for example increasing allocation ratios to more promising parts of the treatment-response relationship, early work (simulations performed but not published as part of [2]) suggested this might not lead to substantial improvements for ROCI trials.

Standard recommendations for MAMS trials apply, with simulations the preferred tool to explore the impact of adaptive steps on the operating characteristics of the trial.

## Implementation: REFINE-Lung

This section illustrates considerations for the design of REFINE-Lung (NCT05085028) with respect to the points in "Design considerations" section, aiming to optimise the frequency of pembrolizumab administration. Pembrolizumab is administered every 6 to 18 weeks and the population of interest is patients with NSCLC who have been on 6-weekly treatment with no progression for six months. The primary outcome is 2-year overall survival; this was chosen as the most clinically significant outcome, given that treatment is offered for two years by the NHS.

### Treatment–response model

While designing REFINE-Lung, it was not possible to find any relevant information to predict the relationship between frequency and response. We therefore use fractional polynomials as a flexible modelling strategy. Specifically, we use “FP2-Select” because, while the frequency-response relationship is likely to be monotonic, it could be flat or more extended frequencies might perform slightly better if, due better tolerability, patients in the reduced intensity arms continued treatment more often, leading to better effectiveness overall. 

### Target scenarios

In terms of the frequency–response relationship, given that the primary endpoint is overall survival, and hence any loss of effectiveness would be unacceptable, we consider a target scenario where all frequencies were equivalent, i.e. a flat frequency-response relationship. 

### Number and position of arms

The standard frequency is 6-weekly, with 18-weekly considered the most extended permissible frequency based on phase I data suggesting drug binding to its target receptor is stable for up to around this period. As many patients receiving immunotherapy treatment receive concurrent chemotherapy given on a 3-weekly administration schedule, it is logical to restrict trial arms (and to a lesser extent subsequent recommendations) to frequencies that are multiples of 3. Hence, we consider five arms: 6, 9, 12, 15 and 18-weekly.

### “Optimality” criterion

We regard the most extended frequency non-inferior to 6-weekly as optimal. We choose survival ratio as the population-level summary measure, i.e. we seek the most extended frequency non-inferior to 6-weekly within a certain margin defined on the survival ratio scale. Aggregating the results of the pembrolizumab trials in NSCLC patients published recently [17–19], a survival ratio margin of 0.88 (same across treatment levels) would preserve 50% of the effect. Hence, the trial will aim to conclude that it is highly likely that more than 88% of the patients that would survive 2 years with a 6-weekly frequency will do so even with a reduced frequency or, in other words, that if 65% of patients survive with the standard frequency, we can rule out that as little as 65*0.88 = 57% of patients or less do so with a reduced frequency.

The main intercurrent event to be expected is patients re-escalating to the standard frequency on progression. As our approach is to evaluate the effectiveness of a strategy of administration of pembrolizumab that involves re-escalation on progression, rather than the actual efficacy of the various frequencies, our main analysis will use a treatment policy strategy for handling this event, and so the analysis will include all randomised individuals.

### Power definition

We explore both possible definitions of power, because only frequencies in multiples of three weeks are relevant pragmatically, so both optimal and acceptable power are meaningful concepts. We define acceptable power here as the probability to identify a frequency within 3-weekly from the true optimum. For acceptable power, a level of 80% is deemed acceptable (no pun intended). For optimal power, we similarly target 80%, although an even lower level could have been more appropriate, given the stringency of its definition and considering the additional “power” stemming from the other non-optimal arms. We decided against this because we feared reviewers would not be ready to accept lower levels, because of the novelty of the method.

### Multiplicity adjustment

No multiplicity adjustment was considered necessary, as the relationship between frequency and response is considered likely to be monotonic and the goal of the trial is to seek the least intensive treatment level non-inferior to 6-weekly. 

### Sample size calculation method

We use the samplesize.ROCI.binary function in the R package {dani} to perform the sample size calculation based on the assumptions and choices above, specifically: 

In the output, it is possible to read the sample size needed to achieve 80% optimal power: 

Power type: optimal power for the following arm(s): 18.

Expected loss to follow-up: 5%.

Total sample size (across all arms): 1803 (95% Monte-Carlo CI: [1648, 1964]).

This estimate is very close to that originally obtained for the trial by simulation (1,750). While this was originally considered achievable, problems with recruitment led us to investigate alternative options, e.g. targeting acceptable power instead. Re-running the above script for power.type=”acceptable” and power.arms = c [15, 17], and increasing loss to follow up to 10% reflecting observed data, returns a more achievable sample size of 1,056 [965, 1,150], close to the 1,100 estimated by simulation. Note that the output returns the Monte Carlo confidence intervals as well, allowing one to decide whether to re-run for larger M.boot, as would be recommended in the example above where the interval is still quite wide. The small M.boot was chosen for illustrative purposes only, as running time for the above code was already around 9 min, although this could have been significantly reduced by using parallel computing (option parallel=”snow” for Windows).

The code below can be used to run simulations, which we used to validate the estimated sample size: 

Even though we only ran a small number of repetitions, to maintain running time as low as one minute, and Monte Carlo error is still large, the simulations confirm optimal power is around 80%: 

expected relationships and NI margins is:

Optimal power: 84% (95% Monte-Carlo CI: [76.81465%, 91.18535%])

Acceptable power: 97% (95% Monte-Carlo CI: [93.65655%, 100%])

Re-running with a larger number of repetitions (1000) returns a 95% Monte-Carlo CI of [80%, 84%]. Of note, power would be slightly lower (and more precise) if using bootstrap within the simulations, but this would be computationally unfeasible.

### Adaptive steps

As the currently recommended frequency is 6-weekly, and as pembrolizumab is an effective drug for patients with NSCLC, immediately opening all 5 frequency arms may be seen as unethical because, although we assumed a flat frequency–response relationship under the alternative hypothesis, the true relationship is of course unknown, and is it possible that lower frequency arms lead to poorer outcomes. We therefore introduced an adaptive element into the design, initially opening only 6 and 12-weekly arms, in order to evaluate whether there was a significant reduction in efficacy with 12-weekly therapy at an interim analysis. Once it was established that 12-weekly therapy was not significantly worse, we later proceeded to open all other arms. Since 2-year overall survival would take too long to accrue for an interim analysis, we based this test on progression-free survival and used a Cox model to estimate the hazard ratio, given that for this 2-arm comparison this had a power advantage.

The original plan was to do this interim analysis within approximately 18 months of the start of recruitment, in order to guarantee that only a certain fraction of total recruitment target in these arms would be achieved by that time. We compared the power of such a comparison being made after a certain number of events in the control arm had been achieved, under different assumptions for the experimental arm event risk. Note that, when implementing such a strategy, in the final analysis a binary indicator of trial phase (i.e. which arms were open) would have to be included to account for the different randomisation arms before and after the interim analysis. 

### Final design

Table 1 summarises the choice to be made in general for a ROCI trial and the ones made specifically for REFINE-Lung.

**Table 1:** Summary of design decisions to be made for (i) a standard 2-arm non-inferiority trial, (ii) a multi-arm non-inferiority trial, (iii) a generic ROCI trial and (iii) REFINE-Lung

	Generic 2-arm Non-inferiority trial	Generic multi-arm Non-inferiority trial	Generic ROCI trial	REFINE-Lung
Treatment-response relationship model	NA	NA	In absence of subject-matter knowledge in favour of a model, FP flexible option. Two choices: maximum # of powers (1: monotonic, less flexible. 2: non-monotonic, more flexible) and type of algorithm (fixed: fixed # of powers. Select: only use more powers when evidence available.	FP2-select chosen as the favoured model. No prior information favouring specific model. Expected monotonicity, but not sure, and prefer more flexibility.
Target scenarios for power	Often same risk in control and active arm	Often same risk in all arms	Often flat treatment-response relationship; either one scenario, or multiple likely ones.	Flat relationship, with survival probability fixed at 65%.
Number and position of arms	Usually standard-of-care and one reduced intensity arm. Hard to pick “right” reduced arm	Usually standard-of-care and some reduced intensity arms. Tricky to pick right number and position of reduced arms.	At least as many arms as parameters in the model. In absence of information, equidistant arms, if clinically meaningful, should be favoured.	6-weekly (standard-of-care), 9-weekly, 12-weekly, 15-weekly and 18-weekly (most extreme frequency suggested by phase 1 data).
“Optimality” criterion	NA	NA	Often minimum treatment intensity leading to acceptable outcome.	Minimum frequency non-inferior to 6-weekly within 0.88 survival ratio margin
Power definitions	Standard definition, usually target levels are 80–90%	Usually familywise power, with target levels 80–90%	Either optimal or acceptable power. 80% might be ok for acceptable power, but lower values for optimal.	Initially 80% optimal power, later revised to 80% acceptable (acceptable frequencies 15 to 18-weekly)
Multiplicity adjustment	NA	Generally required, either Bonferroni correction or some more sophisticated method	Generally not needed as answering single question, in particular if using gatekeeping strategy.	No adjustment needed.
Sample size calculation method	Standard formula, assuming either score or Wald test	Standard formula, assuming either score or Wald test	Either formula based on expected data set, or simulations.	Initially used simulations, later confirmed results with formula.
Adaptive steps	NA	May open more extreme arms only if no evidence of harm, or close them in presence of evidence.	May open more extreme arms only if no evidence of harm, or close them in presence of evidence.	Initially open 6-weekly and 12-weekly only. Later extend to other arms in absence of evidence of harm.

When targeting 80% optimal power, a total sample size of approximately 1,750 patients appears adequate, allowing for 5% loss to follow-up. This number was obtained through simulation, and the calculations through the approximating formula above returned a very similar answer. This was initially regarded as an achievable sample size and selected as the target. This was re-assessed in order to accelerate time to trial completion. Targeting 80% acceptable power, with the range of acceptability being [15-weekly; 18-weekly], the sample size under the same assumptions (but allowing for 10% loss of follow-up) would be reduced to ~1,100 patients. Therefore, this was the new sample size after a major protocol amendment.

For the interim analysis, a sample size of approximately 150 patients provides 80% power to detect a difference of 20 percentage points between the two arms, using a 5% two-sided confidence interval for the hazard ratio for progression-free survival. Of note, the absolute risk difference between patients receiving pembrolizumab or not in the clinical trials was in the order of 30 percentage points, and hence this interim analysis is powered to detect a loss of efficacy of about two thirds.

## Discussion

We have described the decisions that researchers need to take when planning a trial with a ROCI design. We have illustrated how the proposed strategy was used to plan the REFINE-Lung trial, whose aim is to optimise immunotherapy treatment frequency for patients with NSCLC. 

### Recommendations

1. When the research goal is to optimise some aspect of treatment administration that can be seen as (nearly-)continuous, i.e. duration, dose or frequency, a ROCI trial is a valid option.
2. It is important to explain and justify the following choices: model for the treatment-response relationship, number and position of arms, “optimality” criterion, power definition, target scenarios, sample size calculation method and possibly adaptive steps.
3. Fractional polynomial models with 2 powers are often a good flexible choice, either implemented as in {mfp}’s default (when monotonicity is likely) or fixing the number of powers. At least 5 arms should be used (ideally 6 or 7), and these should be equally spaced if practicable.
4. The “optimal” treatment is usually the least intensive level leading to good outcomes; it is possible to power the trial to detect such optimal treatment level (i.e. optimal power) but may be unfeasible. A more practically viable approach is to power to detect a treatment level within a certain acceptable range from the optimal (i.e. acceptable power).
5. Powering assuming a flat treatment–response relationship is equivalent to standard non-inferiority trials; however, if feasible and backed by existing data/knowledge, particularly regarding the relative importance of preserving effectiveness of the primary outcome vs. secondary benefits from reducing administration, it is possible to use different target scenarios.
6. For binary outcomes, the sample size calculation formula presented in (Bern et al.) can be used for specific assumptions, or simulations can be used.

### Extensions

While the current methodology has been developed for binary outcomes, it can be readily extended to continuous and time-to-event data, and we are currently working on investigating the impact of using different outcome types.

We plan to investigate how to incorporate additional variables in the design, either simply as adjustment variables to gain power, or with interactions, to perform subgroup analyses and possibly give different recommendations to different groups.

Finally, Bayesian methods could be considered particularly appealing for ROCI designs, as the focus is more on modelling than on answering a single binary question and this would allow one to interpret the posterior probability of a treatment level being non-inferior. Possible extensions to design a ROCI trial in the Bayesian framework might follow.

## Conclusions

The (MAMS-)ROCI, aka DURATIONS, randomised trial design is a novel alternative to standard two arm non-inferiority designs for trials that seek to optimise ordinal aspects of treatment administration, such as duration, dose or frequency. We have shown how such a trial can be designed and illustrated this using the REFINE-Lung trial, which may act as an example for future trials using a ROCI design.

## Data Availability

No datasets were generated or analysed during the current study.
